# Identification of immune‐related genes contributing to head and neck squamous cell carcinoma development using weighted gene co‐expression network analysis

**DOI:** 10.1002/cnr2.1808

**Published:** 2023-04-24

**Authors:** Qiaojuan Guo, Tianzhu Lu, Hanchuan Xu, Qingfeng Luo, Zhiliang Liu, Sicong Jiang, Jianji Pan, Shaojun Lin, Mengyao Lin, Fang Guo

**Affiliations:** ^1^ Key Laboratory of Systems Biomedicine (Ministry of Education), Shanghai Center for Systems Biomedicine Shanghai Jiao Tong University Shanghai China; ^2^ Department of Radiation Oncology Fujian Medical University Cancer Hospital, Fujian Cancer Hospital Fuzhou Fujian China; ^3^ Department of Radiation Oncology Jiangxi Cancer Hospital Nanchang China; ^4^ National Health Commission Key Laboratory of Personalized Diagnosis and Treatment of Nasopharyngeal Carcinoma, Jiangxi Cancer Hospital Nanchang China; ^5^ Department of Pathology Jiangxi Cancer Hospital Nanchang Jiangxi China; ^6^ Division of Thoracic and Endocrine Surgery University Hospitals and University of Geneva Geneva Switzerland

**Keywords:** biomarkers, differentially expressed genes, head and neck squamous cell carcinoma, PPI network analysis, WGCNA

## Abstract

**Background:**

This study aimed to identify genes related to the degree of immune cell infiltration in head and neck squamous cell carcinoma (HNSCC), explore their new biological functions, and evaluate their diagnostic and prognostic value in HNSCC.

**Methods:**

Transcriptomic data from The Cancer Genome Atlas (TCGA) HNSCC dataset was used to screen differentially expressed genes between tumors and normal tissues, followed by weighted correlation network analysis (WGCNA) to identify immune‐related modules. Differential gene expression, immune cell infiltration, and survival analyses were performed to screen key genes. The expression of these key genes was validated in Oncomine and gene expression omnibus (GEO) datasets and by immunohistochemistry (IHC).

**Results:**

1869 and 1578 genes were significantly upregulated and downregulated in HNSCC. WGCNA showed that the brown module was associated with the most significant number of immune‐related genes. PPI network analysis demonstrated that PPL, SCEL, KRT4, KRT24, KRT78, KRT13, SPRR3, TGM3, CRCT1, and CRNN were key components in the brown module. Furthermore, the expression levels of KRT4, KRT78, KRT13, and SPRR3 in HNSCC correlated with infiltration levels of CD8+ T cells and macrophages. Survival analyses revealed that the expression of KRT78, KRT13, and SPRR3 in HNSCC correlated with overall survival (OS). The IHC assay indicated that KRT13 (*p* = .042), KRT78 (*p* < .001), and SPRR3 (*p* = .022) protein expression levels in HNSCC were significantly lower than in normal tissues. Analysis of GSE65858 and GSE41613 datasets showed that a worse OS was associated with low expression of KRT78 (*p* = .0086, and *p* = .005) and SPRR3 (*p* = .017, and *p* = .02).

**Conclusions:**

Our findings suggest that KRT4, KRT78, KRT13, and SPRR3 are related to the occurrence and development of HNSCC. Importantly, KRT78 and SPRR3 might serve as diagnostic and prognostic biomarkers of HNSCC.

## 1 INTRODUCTION

Head and neck squamous cell carcinoma (HNSCC) is a common malignancy, with an annual incidence of 500 000 cases worldwide.^1^, ^2^ It is well‐established that most patients with HNSCC are diagnosed with advanced or metastatic disease at diagnosis, with a 5‐year overall survival (OS) rate of only 50% and a recurrence or metastasis rate of 30%.^3^, ^4^ Indeed, once recurrence and metastasis occur, the prognosis is poor, and the median survival time is only 6 months. These findings emphasize the need to identify novel approaches to reduce recurrence and metastasis rates and improve the survival of patients with recurrent and metastatic HNSCC.

In recent years, tumor immunity research has gathered momentum given the efficacy of immune checkpoint inhibitor (ICI) treatment in various tumors.^5^ An increasing body of evidence suggests that immune cell infiltration is essential for the efficacy of ICIs^6^, ^7^, ^8^ and the cause and predictor of cancer recurrence and metastasis.^6^, ^7^ It is well‐established that ICIs are efficient in HNSCC^8^; however, predicting the efficacy of ICIs and the mechanism underlying ICI resistance remain unclear. Current evidence suggests that the tumor immunosuppressive microenvironment can inhibit immune cell function and infiltration levels.^9^ Accordingly, it is necessary to explore the mechanisms underlying the inhibition of immune cell infiltration in HNSCC.

The rapid advent of bioinformatics has enhanced our understanding of the biological functions of tumors, providing the foothold for the discovery and verification of tumor biomarkers. There is ample evidence showing that immune‐related genes in HNSCC can predict tumor prognosis and are potential biomarkers.^10^, ^11^ However, these genes are known to be inversely related to immunity. Importantly, bioinformatic analysis provides a novel approach to uncover genes related to immune cell infiltration and explore their biological functions and their potential clinical translational value as diagnostic and prognostic markers.

Herein, weighted correlation network analysis (WGCNA) was conducted to identify highly correlated gene clusters using module clustering.^12^ We sought to identify specific immune‐related genes associated with the development or prognosis of HNSCC from The Cancer Genome Atlas (TCGA). Furthermore, we explored key genes related to the occurrence of HNSCC, the biological process involved, and their prognostic value in this patient population.

## 2 MATERIALS AND METHODS

### 2.1 Data collection and preprocessing

The TCGA (https://tcgadata.nci.nih.gov/tcga/) HNSCC transcriptomic and clinical data, and two Gene Expression Omnibus (GEO) datasets (GSE65858 and GSE41613) were downloaded. A total of 528 HNSCC patients were identified in the TCGA database, consisting predominantly of males (73.11%) with no previous history of cancer (94.89%). We used DESeq2^13^ to analyze the differential expression of genes in the transcriptomic data of TCGA HNSCC database. The screening criteria for differentially expressed genes (DEGs) included: |log2 (fold change)|>2 and *p* < .05.

In addition, the expression levels of key genes of two HNSCC datasets from the Oncomine database (Ye Head‐Neck,^14^ and Peng Head‐Neck),^15^ were included in our analyses.

### 2.2 Weighted correlation network analysis

WGCNA^16^ was used to identify co‐expressed gene modules in DEGs and convert the adjacency matrix into a topological overlap matrix (TOM). According to the TOM‐based dissimilarity, DEGs were finally divided into 13 different modules. Using the InnateDB^17^ database, a database of genes involved in innate immunity, we identified 824 immune‐related genes, of which 72 exhibited significant changes in HNSCC. Furthermore, genes from the brown module were selected as the gene set for subsequent analysis since it contained the most significant number of immune‐related DEGs (*n* = 223).

### 2.3 Gene ontology function enrichment analysis

Metascape^18^ (https://metascape.org/), an online analysis tool, was used to perform Gene ontology (GO) function enrichment analysis (Biological Process, Molecular Function, Cellular Component) on DEGs, with the parameters set to min overlap = 3, *p*‐value cutoff = .01, and min enrichment = 1.5.^19^ Specifically, the immunological signature (Immunologic Signature) was selected for functional enrichment analysis, and the parameters remain unchanged.

### 2.4 Gene set enrichment analysis

The Gene set enrichment analysis (GSEA)^20^ method was used to screen the biological state or process related to the occurrence of HNSC. We selected “h.all.v7.1.symbols.gmt” as the reference set, the number of permutations was set to 1000, and the permutation type set to the selected gene set. Gene sets with an FDR of <0.05 were significantly enriched, and the Normalized Enrichment Score (NES) was used to indicate the degree of enrichment.

### 2.5 Protein–protein interaction network construction

We obtained the protein–protein interactions (PPI) (score >0.4) of 223 immune‐related DEGs using STRING database^21^ (https://string-db.org/), and the main interaction network was visualized using Cytoscape.^22^ The top 10 key genes were obtained using CytoHubba, and the interaction network was drawn.

### 2.6 Tumor immune infiltration analysis

We analyzed the gene expression data of HNSCC samples from TCGA using TIMER2.0^23^ and analyzed the correlation between KRT4, KRT78, KRT13, and SPRR3 expression and immune cell (CD8+ T cells and macrophages) infiltration levels.

### 2.7 Immunohistochemistry

This study was approved by the Review Board Committee of Fujian Cancer Hospital, Fujian, China. The Review Board Committee granted a waiver of informed consent for this study. The informed consent Tissue samples from cases of pathologically diagnosed HNSCC (*n* = 70) treated at the Fujian Cancer Hospital from 2008 to 2017, and normal tissue samples (*n* = 10) were included in the analysis. The clinicopathological features of HNSCC and normal samples are shown in Table 3. The samples were fixed in formaldehyde and processed with heat‐mediated antigen retrieval in citrate buffer (pH = 6). The samples were then blocked and incubated with the following primary antibodies: rabbit polyclonal anti‐KRT13 (1:1000, Cat No. A116809, SIGMA Life Science, USA), rabbit polyclonal anti‐KRT78 (1:1000, 000015851, SIGMA Life Science, USA), and rabbit polyclonal anti‐SPRR3 (1:200, Cat No. ab218131, Abcam, USA) at 4°C overnight. The ElivisionTM plus Polyer HP (Mouse/Rabbit) immunohistochemistry (IHC) Kit (Cat. KIT‐9901, MXB biotechnologies, China) was used for IHC detection. Two independent pathologists, blinded to the clinicopathological data, evaluated the immunohistochemical score.

### 2.8 Statistical analysis

The Wilcoxon rank‐sum test and Wilcoxon signed‐rank test were used to analyze the expression of key genes in tumor and normal tissues. The relationship between clinicopathological features and KRT4, KRT78, KRT13, and SPRR3 expression was assessed by the Kruskal–Wallis test, Wilcoxon signed‐rank test, and chi‐squared test. Survival curves were drawn using the Kaplan–Meier method, and differences between groups were assessed using the log‐rank test. OS was defined as the time of diagnosis until the date of death from any cause or last follow‐up. Progression‐free survival (PFS) was defined as the time of diagnosis until the date of disease progression, death or last follow‐up. A *p*‐value <.05 (two‐sided) was statistically significant. Statistical analyses were carried out using R (version 3.6.1) and SPSS (version 24.0).

## 3 RESULTS

### 3.1 Identification and functional enrichment analysis of DEGs related to the occurrence of HNSCC

**FIGURE 1:**
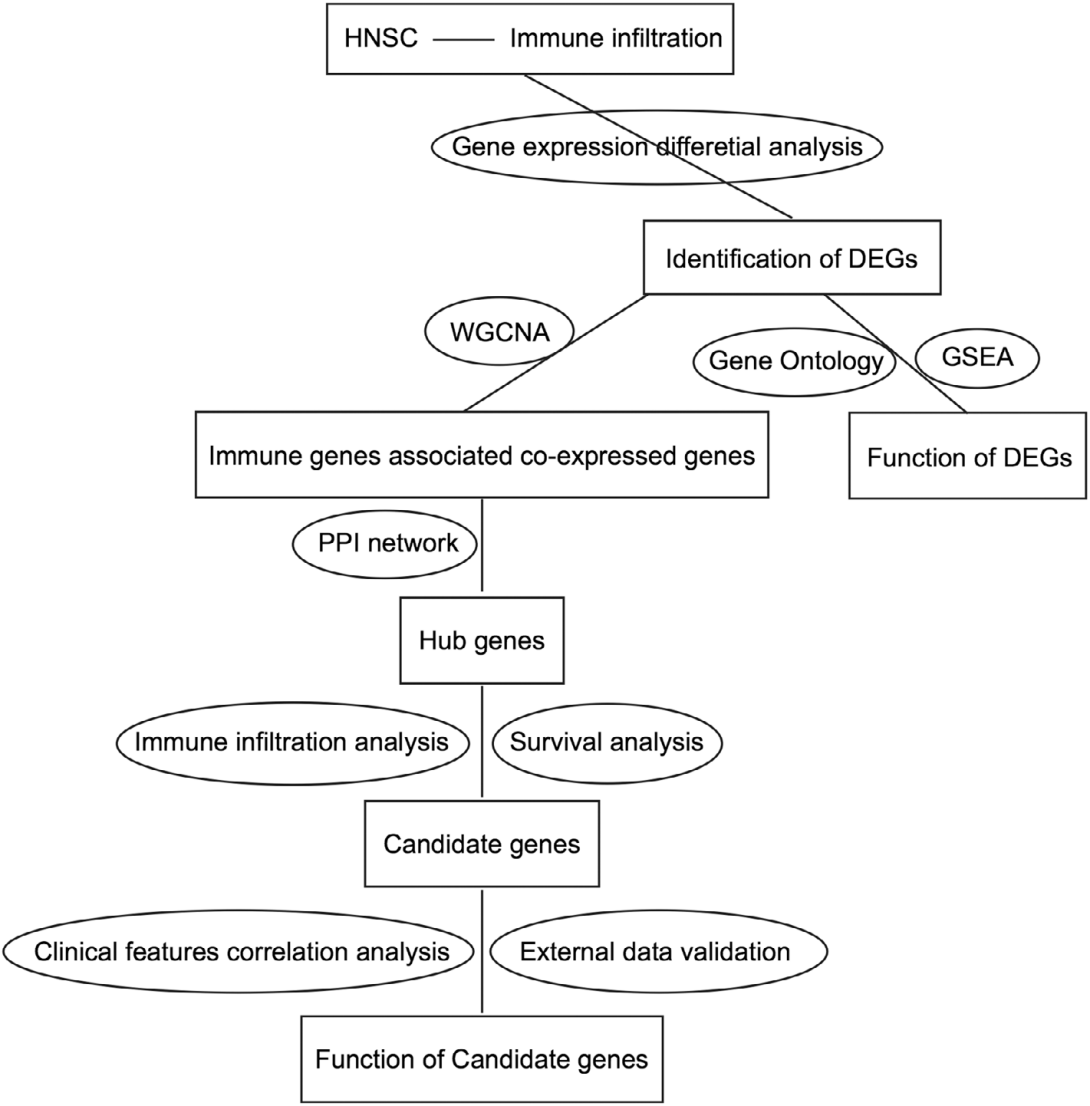
Flow chart of overall analysis

**FIGURE 2:**
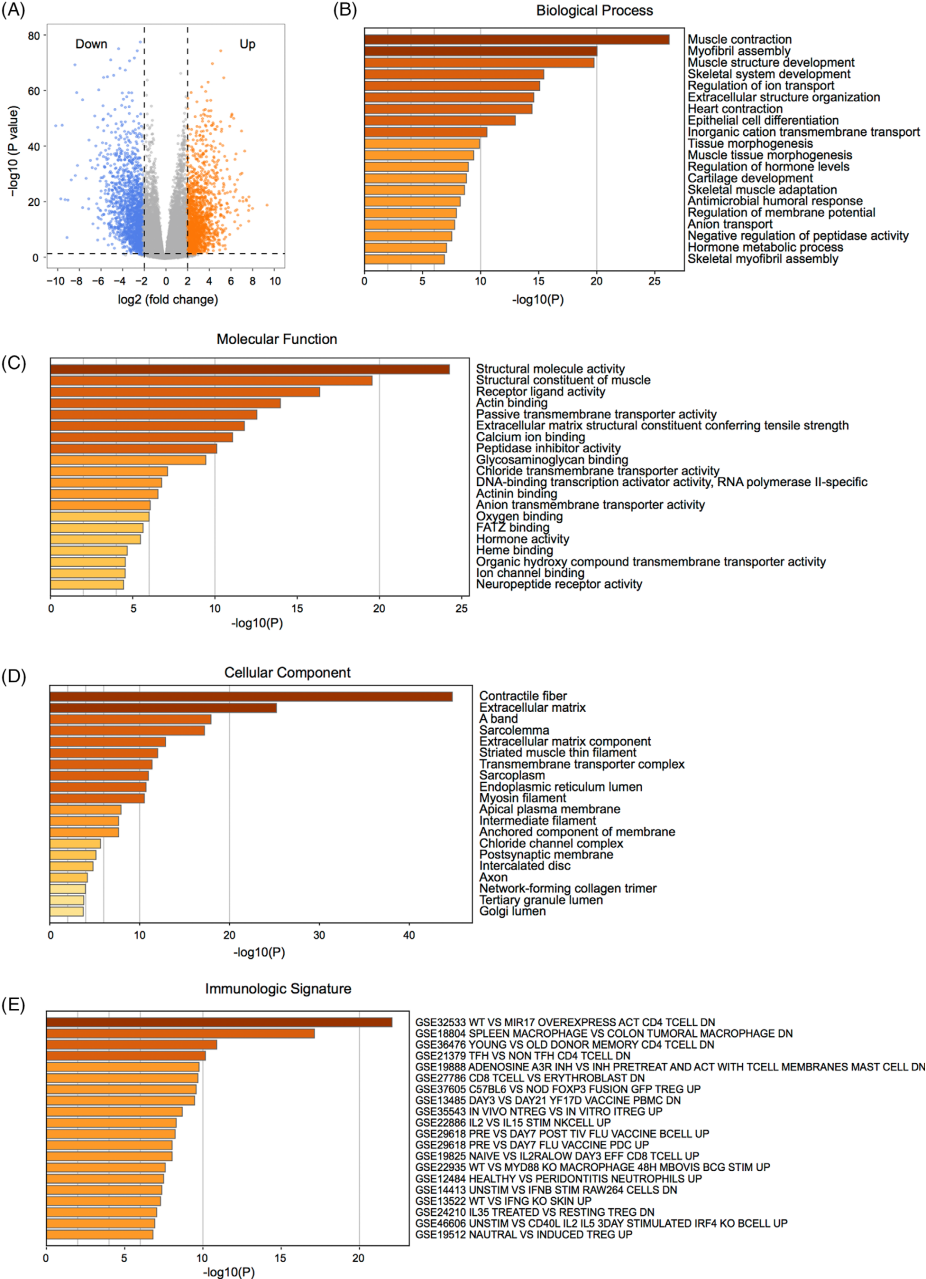
Identification and functional enrichment analysis of differentially expressed genes in head and neck squamous cell carcinoma (HNSCC)

To explore the relationship between HNSCC development and immune infiltration, we conducted a comprehensive integrated analysis of transcriptomic and clinical data of HNSCC patients from TCGA (Figure 1, Table 1). First, differential expression analysis showed that 1869 and 1578 genes were significantly upregulated and downregulated in HNSCC, respectively (Figure 2A, Table S1).

**TABLE 1:** Clinical characteristics of patients in the The Cancer Genome Atlas database

Clinical characteristics	Number (%)
Gender	
Male	386 (73.11)
Female	142 (26.89)
Prior malignancy	
Yes	27 (5.11)
No	501 (94.89)
Vital status	
Alive	304 (57.58)
Dead	224 (42.42)
AJCC clinical T	
T1	37 (7.01)
T2	152 (28.79)
T3	139 (26.33)
T4	184 (34.85)
Not reported	16 (3.03)
AJCC clinical N	
N0	246 (46.59)
N1	85 (16.10)
N2	166 (31.44)
N3	9 (1.70)
Not reported	22 (4.17)
AJCC clinical M	
M0	496 (93.94)
M1	6 (1.14)
Not reported	26 (4.92)
AJCC clinical stage	
I	21 (3.98)
II	99 (18.75)
III	107 (20.27)
IV	287 (54.36)
Not reported	14 (2.65)

**FIGURE 3:**
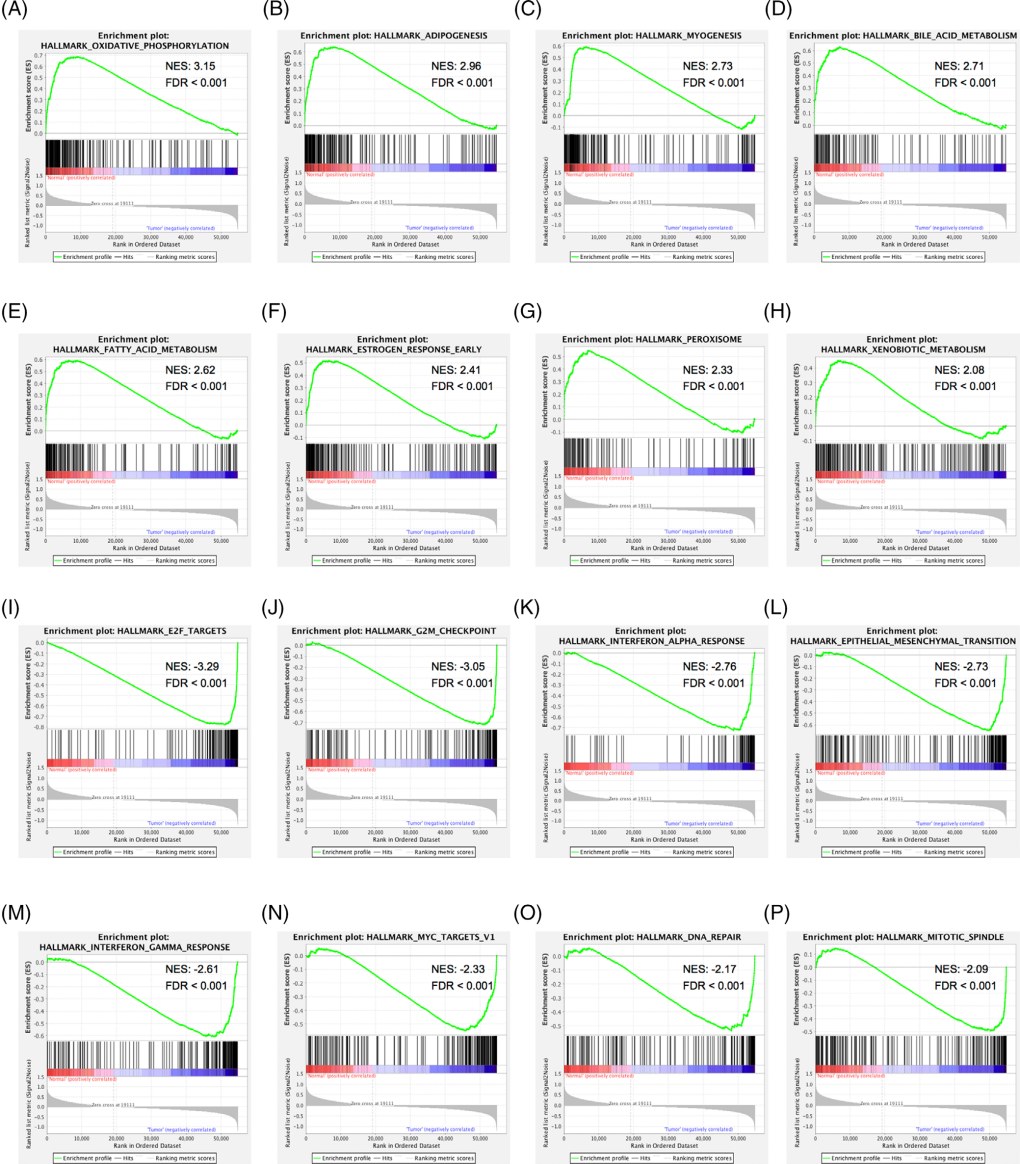
Gene set enrichment analysis (GSEA) of differentially expressed genes (DEGs) related to head and neck squamous cell carcinoma

GO annotation demonstrated that these DEGs were mainly enriched in biological processes, including muscle contraction, myofibril assembly, and muscle structural development (Figure 2B). GO terms for molecular functions consisted of structural molecular activity, muscle structural components, and receptor ligand activity (Figure 2C), and the related cellular components included contractile fibers, extracellular matrix, and A‐bands (Figure 2D). Meanwhile, enrichment analysis of the immune characteristics of these DEGs showed that they were involved in the regulation of CD4+ T cells, macrophages, and CD8+ T cells (Figure 2E, Table S2). Furthermore, GSEA showed that biological processes that were significantly enriched in normal tissues included oxidative phosphorylation (Figure 3A), adipogenesis (Figure 3B), myogenesis (Figure 3C), bile acid metabolism (Figure 3D), fatty acid metabolism (Figure 3E), early estrogen response (Figure 3F), peroxisomes, (Figure 3G) and xenobiotic metabolism (Figure 3H). The biological processes significantly enriched in HNSCC encompassed E2F transcription factor targeting (Figure 3I), G2M checkpoint (Figure 3J), interferon α response (Figure 3K), epithelial‐mesenchymal transition (Figure 3L), interferon γ response (Figure 3M), MYC targeting (Figure 3N), DNA repair (Figure 3O) and mitotic spindle (Figure 3P; Table S3).

### 3.2 Co‐expression analysis of DEGs and PPI network in HNSCC

**FIGURE 4:**
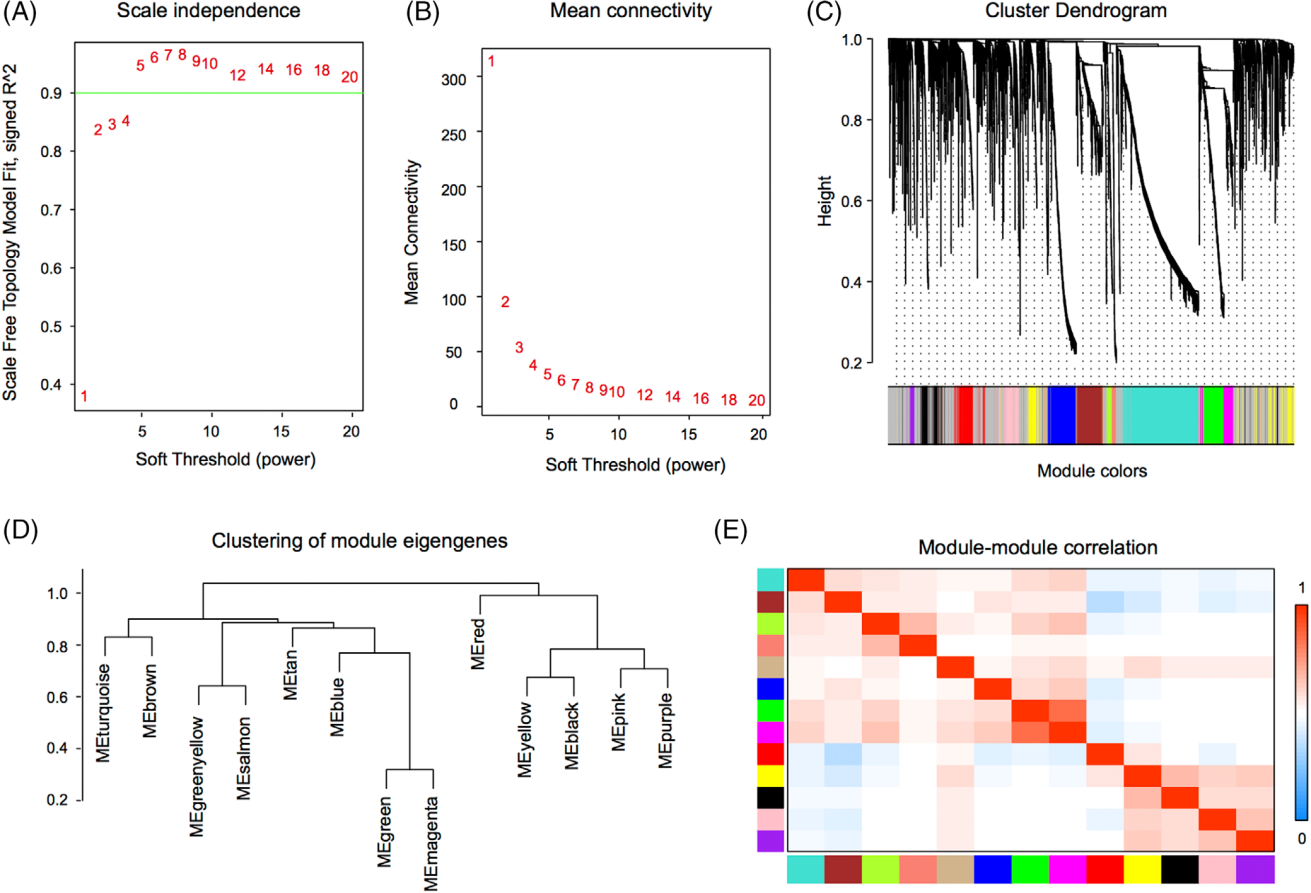
Co‐expression analysis of differentially expressed genes (DEGs) in head and neck squamous cell carcinoma

**FIGURE 5:**
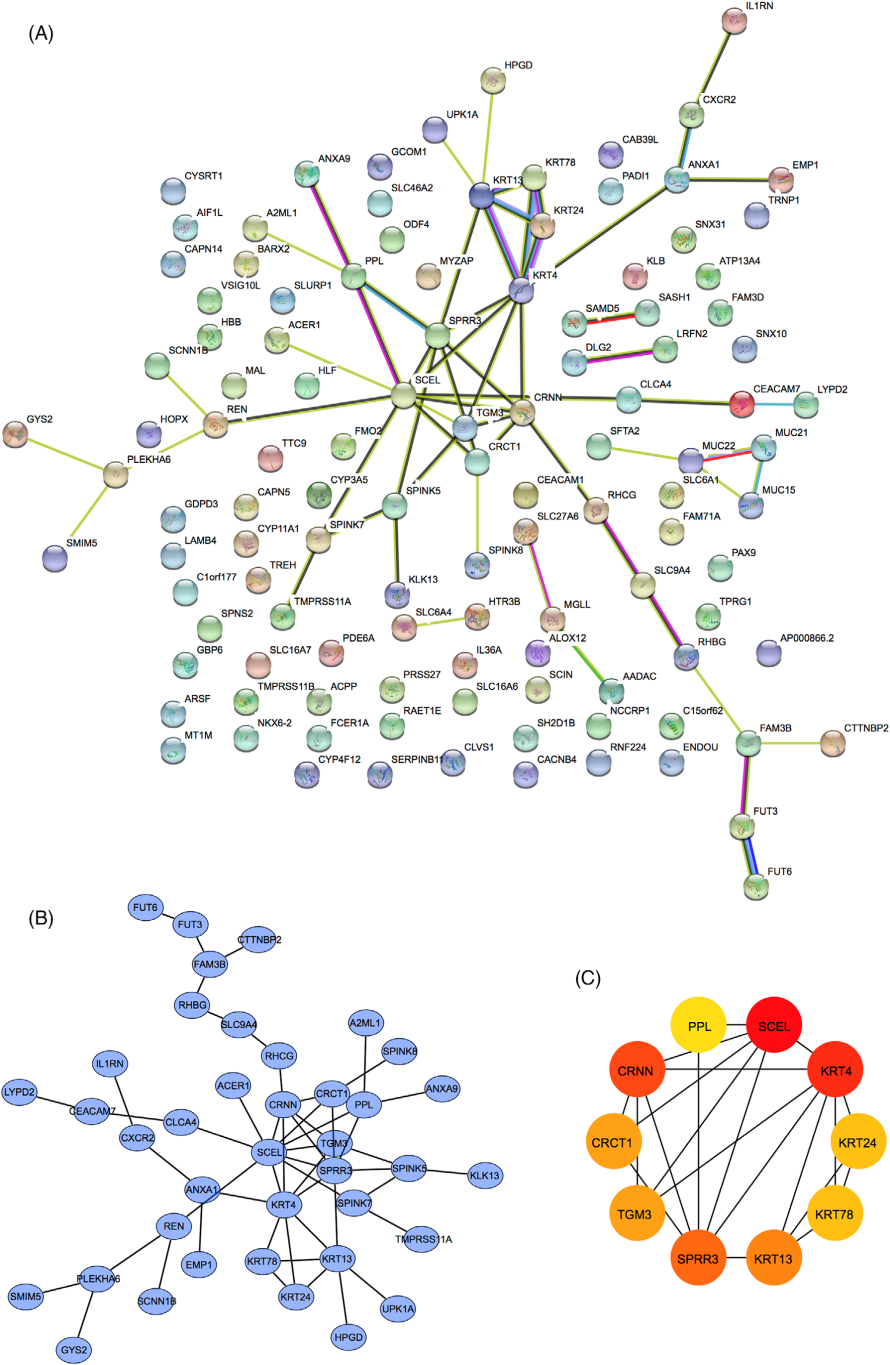
Protein–protein interaction (PPI) network of immune‐related differentially expressed genes (DEGs).

To identify gene sets related to the occurrence of HNSCC, we performed WGCNA based on transcriptomic data. Using a soft‐threshold of 4 (Figure 4A,B), WGCNA identified 13 modules (Figure 4C–E). Compared with the other modules, the brown module contained the most significant number of immune‐related genes (*n* = 7; Table S4). Two hundred and twenty‐three genes were enriched in the brown module, and many interactions were identified between the proteins encoded by these genes (Figure 5A,B). Proteins encoded by PPL, SCEL, KRT4, KRT24, KRT78, KRT13, SPRR3, TGM3, CRCT1, and CRNN were core components in the PPI network (Figure 5C).

### 3.3 Differential expression, immune cell infiltration, and survival analysis of key genes

**FIGURE 6:**
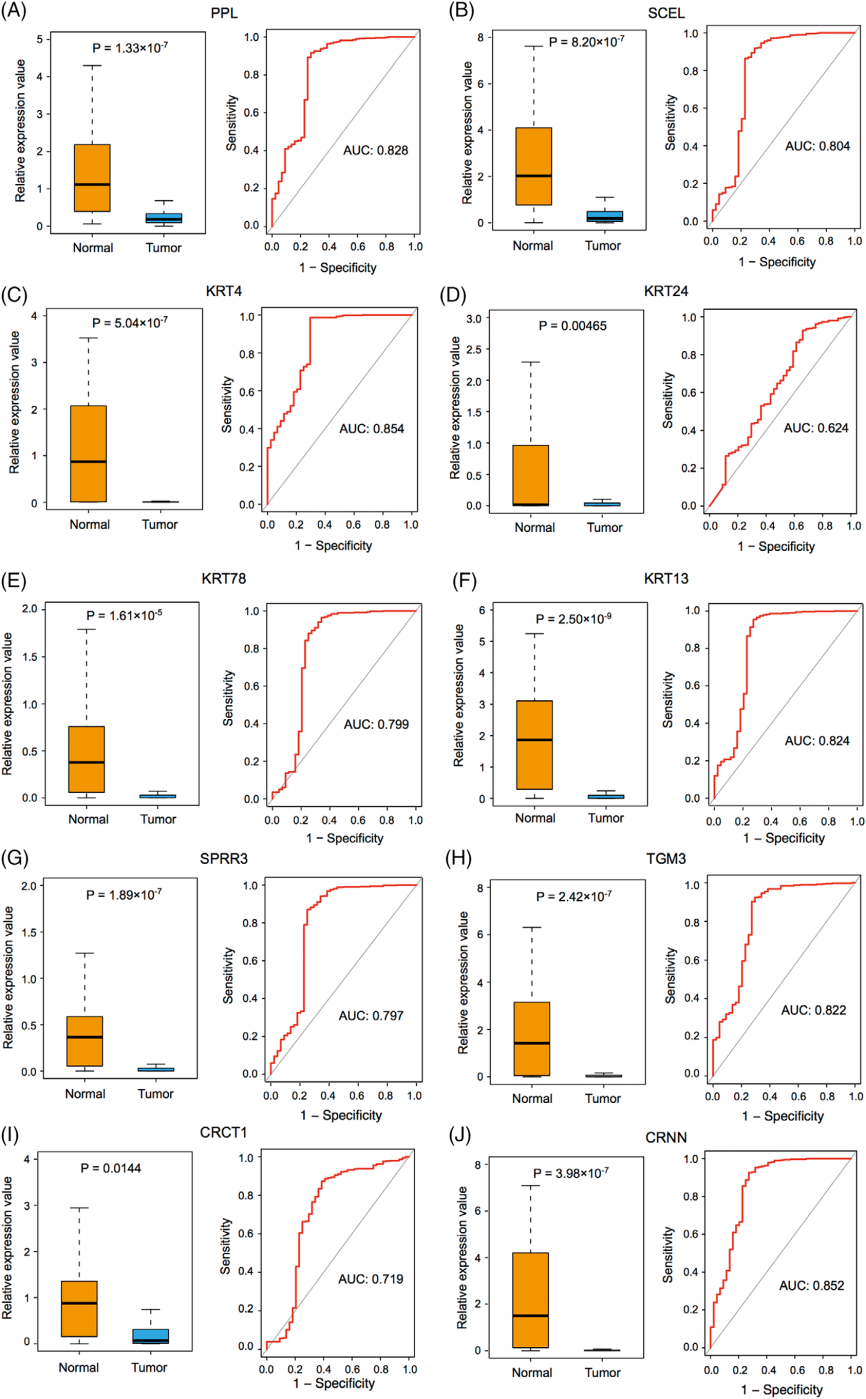
The RNA expression of key genes in head and neck squamous cell carcinoma

**FIGURE 7:**
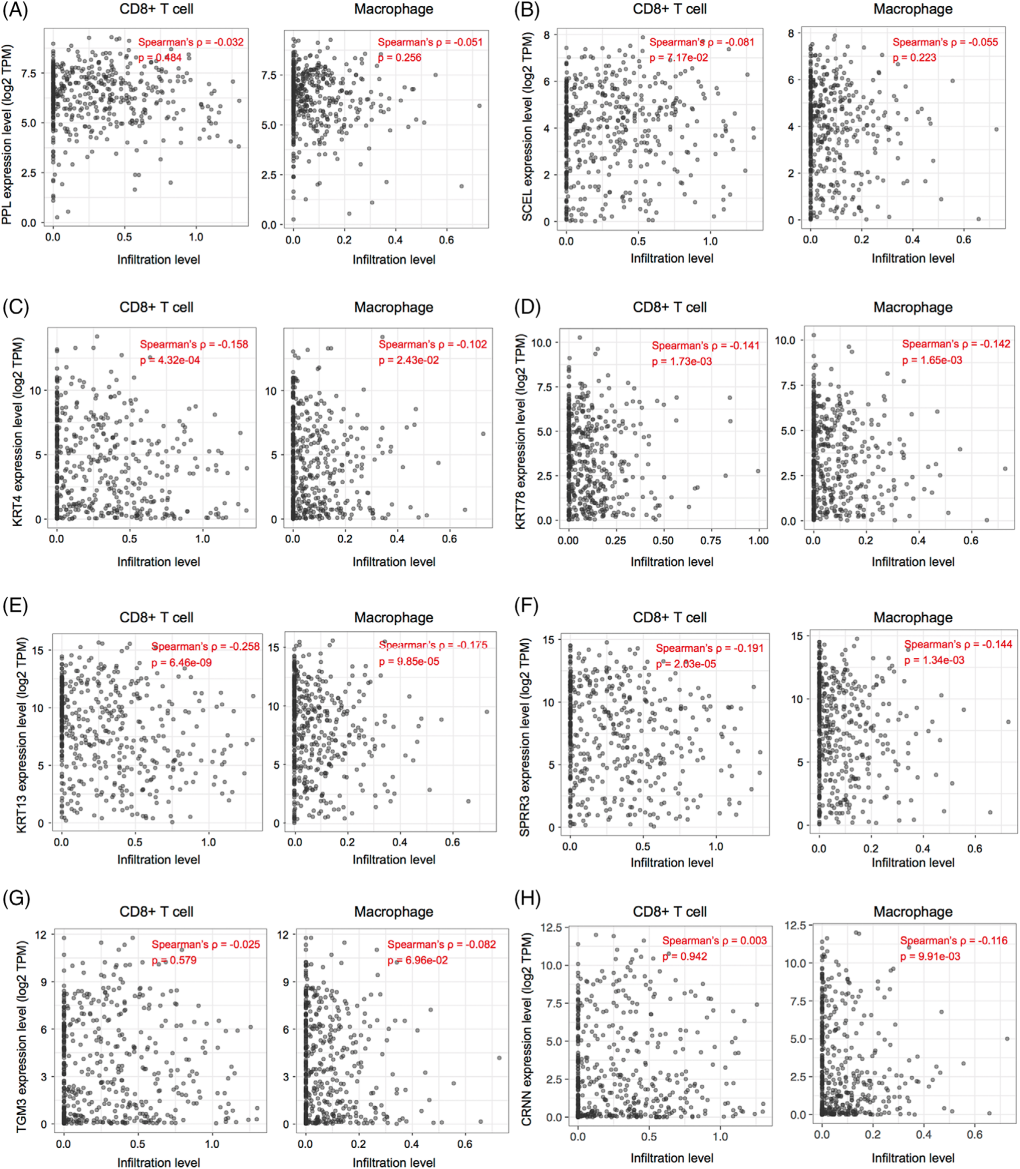
Accurate prediction of the correlation between key genes and immune cell infiltration in head and neck squamous cell carcinoma

**FIGURE 8:**
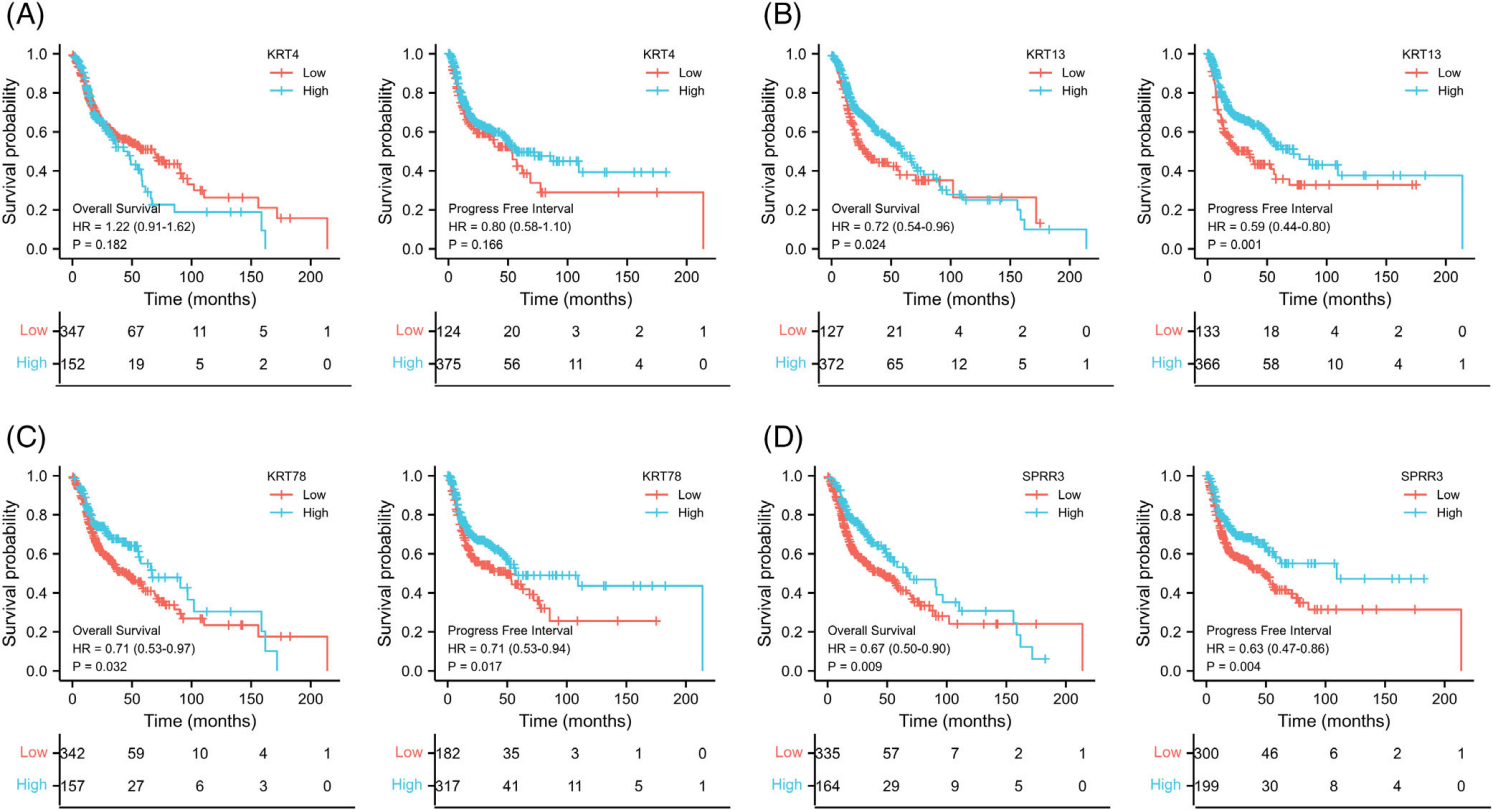
Survival analysis of key genes in head and neck squamous cell carcinoma

The expression of key DEGs screened from normal and HNSCC tissues was analyzed. We found that their expression levels in the HNSCC group were significantly lower than in the normal group (Figure 6A–J, left). In the meantime, we found that except for KRT24 and CRCT1 (AUCs <0.75), the other eight genes yielded a high predictive performance for the occurrence of HNSCC, with AUC values of 0.828 (PPL), 0.804 (SECL), 0.854 (KRT4), 0.799 (KRT78), 0.824 (KRT13), 0.797 (SPRR3), 0.822 (TGM3), and 0.852 (CRNN; Figure 6A–J, right). Further analyses of immune cell infiltration of these eight genes showed that the expression levels of KRT4, KRT78, KRT13, and SPRR3 were significantly correlated with the infiltration levels of CD8+ T cells and macrophages in HNSCC (Figure 7). Then, survival analyses of these four genes were conducted, including OS and DFS, and results showed that except for KRT4, the other three genes were related to OS and DFS in HNSCC (Figure 8A). KRT78 expression was significantly related to OS and DFI (Figure 8B). KRT13 and SPRR3 expression levels were significantly related to DFI (Figure 8C) and OS (Figure 8D), respectively. Finally, KRT4, KRT78, and SPRR3 were selected as our candidate genes.

### 3.4 Correlation analysis of key gene expression and clinical characteristics

**FIGURE 9:**
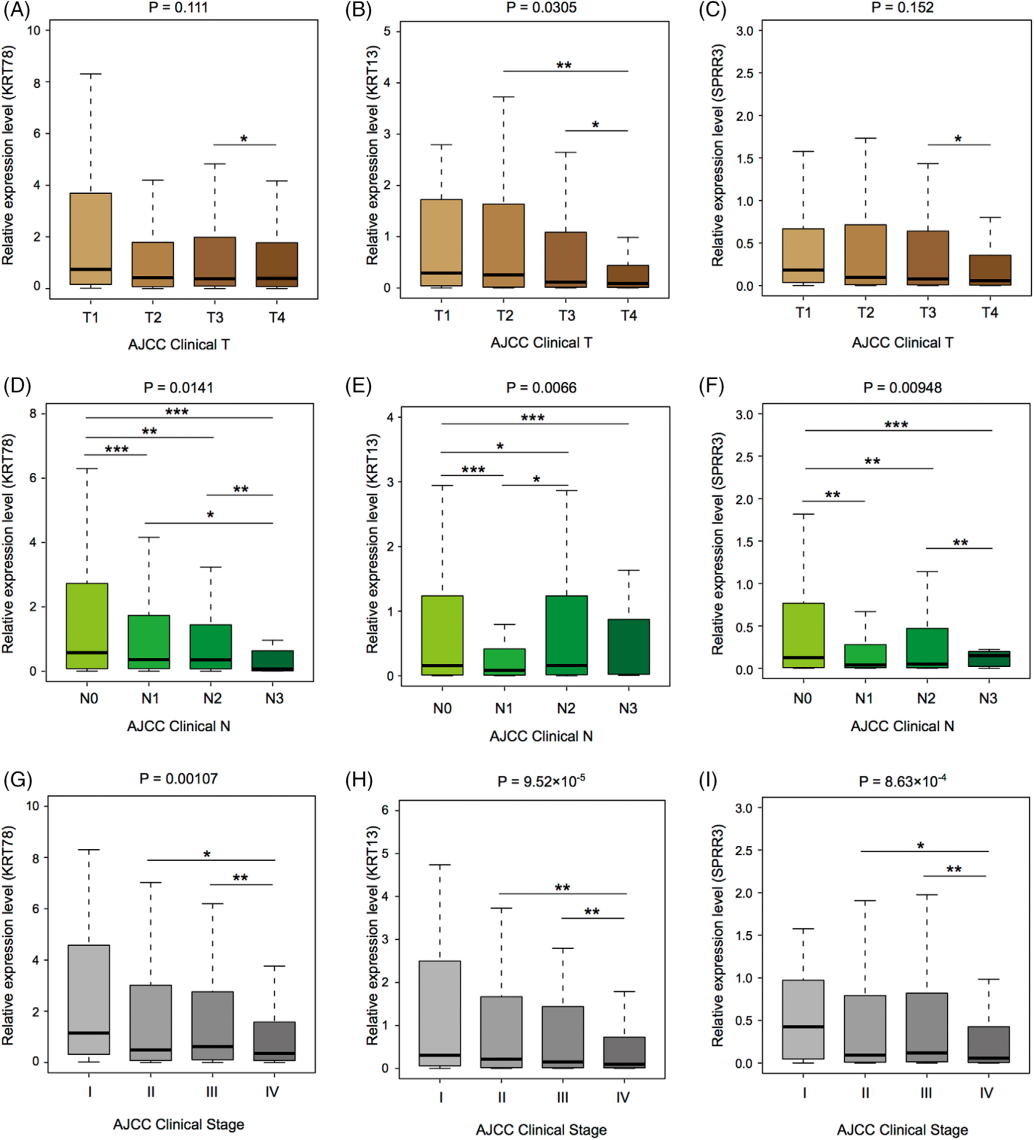
Correlation between the expression level of candidate genes and the clinical stage of head and neck squamous cell carcinoma

A low correlation was found between KRT78 and SPRR3 expression levels and clinical T stage. However, the expression levels of these two genes in T4 HNSCC patients were significantly lower than in T3 HNSCC patients (Figure 9A,B), while no correlation was found between KRT13 expression and T stage (Figure 9C). The expression levels of these three candidate genes showed a decreasing trend as the N stage increased (Figure 9D–F). Similarly, KRT78, KRT13, and SPRR3 expression levels showed a downward trend with increasing HNSCC clinical stage (Figure 9G–I). These results suggested that the three candidate genes were closely related to HNSCC development (Table 2).

**TABLE 2:** Demographic and clinical characteristics of head and neck squamous cell carcinoma (HNSCC) patients in GSE41613 and GSE65858

Characters	GSE41613	GSE65858
	*n* (%)	*n* (%)
Gender		
Female	31 (32.0)	43 (17.4)
Male	66 (68.0)	223 (82.6)
Age (year)		
<60	50 (51.5)	153 (56.7)
≥60	47 (48.5)	117 (43.3)
HPV		
Negative	97 (100)	197 (73.0)
Positive	0 (0)	73 (27.0
T classification		
T1	NA	115(42.6)
T3	NA	155 (57.4)
N classification		
N0‐N1	NA	126 (46.7)
N2‐N3	NA	144 (53.3)
Clinical stage		
I‐II	41 (42.3)	55 (20.4)
III‐IV	56 (57.7)	215 (79.6)
Comprehensive treatment		
No	44 (45.4)	
Yes	53 (54.6)	

### 3.5 External validation of candidate genes

**FIGURE 10:**
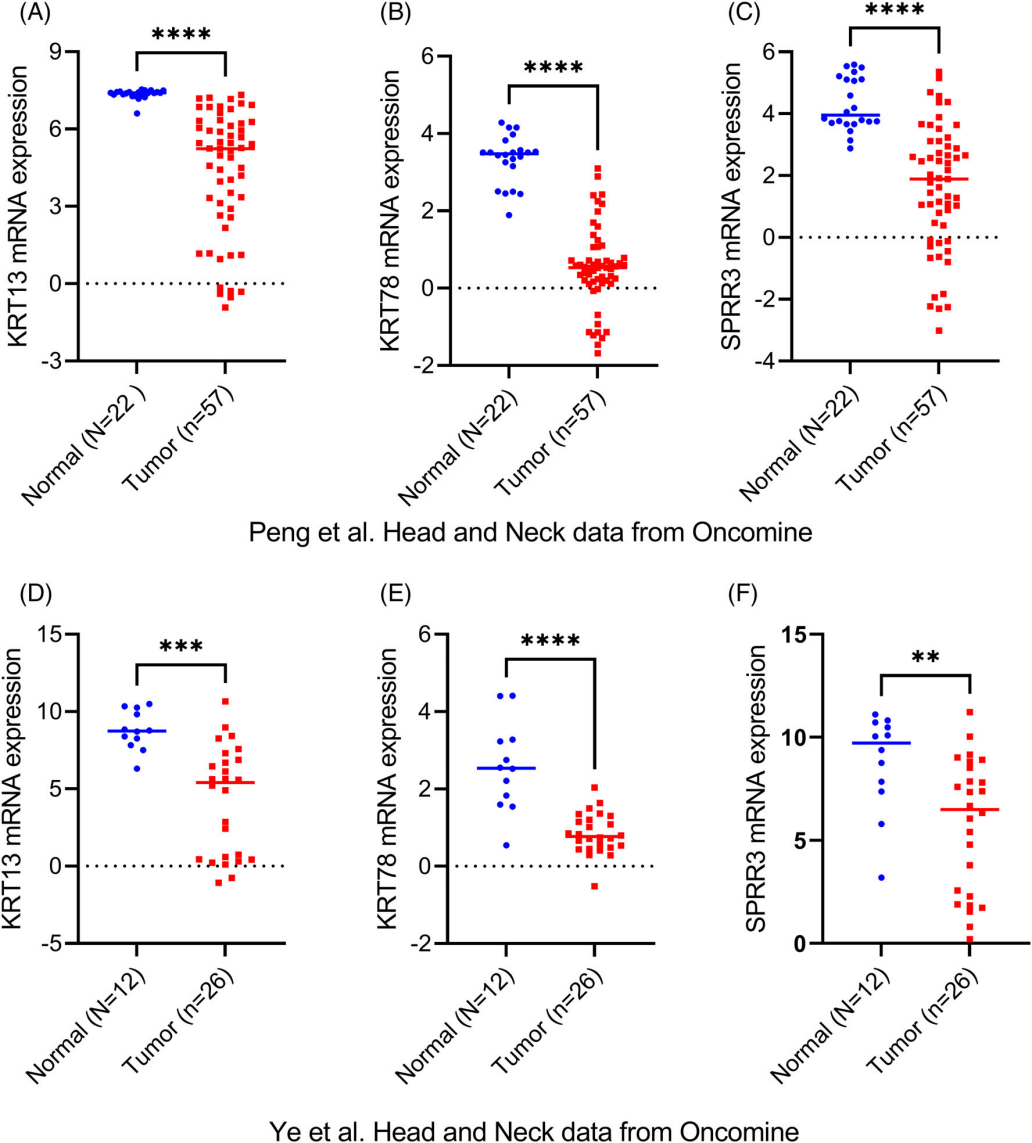
Verification of candidate gene mRNA expression levels in Oncomine datasets

We used Oncomine datasets (Ye Head‐Neck, and Peng Head‐Neck) to verify the expression levels of KRT13, KRT78, and SPRR3 in HNSCC tumor and normal tissues. Analysis of the Ye Head‐Neck (Figure 10A–C) and Peng Head‐Neck (Figure 10D–F) datasets showed that the expression levels of KRT13 (*p* < .001), KRT78 (*p* < .001), and SPRR3 (*p* < .001) in HNSCC were significantly lower than in normal tissues.

**FIGURE 11:**
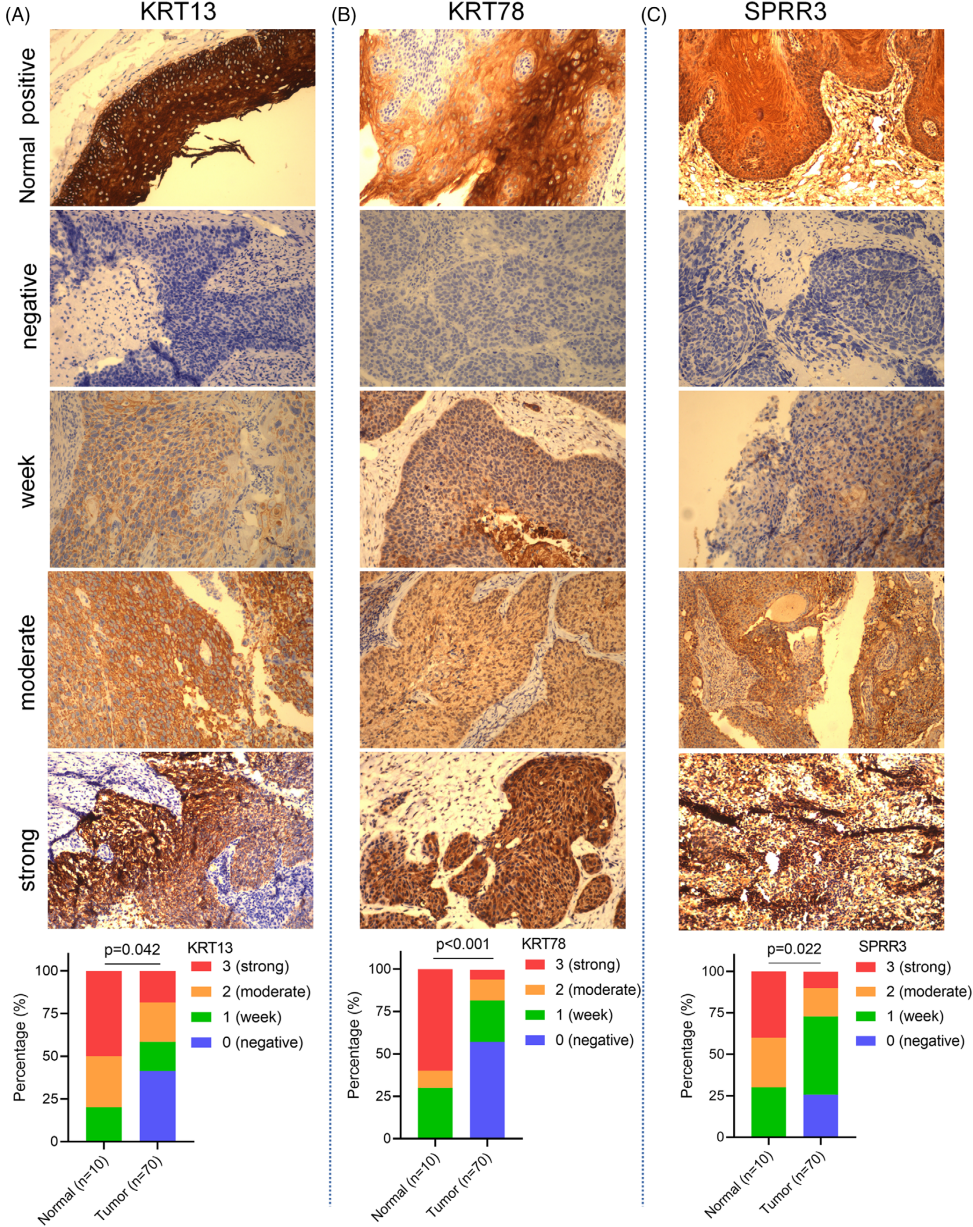
Validation the protein expression of KRT13, KRT78, and SPRR3 in head and neck squamous cell carcinoma tissues and normal tissues by immunohistochemistry

To further analyze differences in the expression of KRT13, KRT78, and SPRR3 genes in HNSCC and normal tissues, we used a microarray of 10 normal tissues and 70 HNSCC tissues to verify the protein expression of the above genes. The results showed that the protein expression levels of KRT13 (*p* = .042, Figure 11A), KRT78 (*p* < .001, Figure 11B), and SPRR3 (*p* = .022, Figure 11C) in HNSCC tissues were significantly lower than in normal tissues (Table 3).

**TABLE 3:** KRT13, KRT78, and SPRR3 protein expression of head and neck squamous cell carcinoma (HNSCC) patients (*n* = 70) and normal tissue (*n* = 10) in validation cohort of protein expression

Characters	Level	HNSCC	Normal tissue	*p*
Gender	Female	5 (7.1%)	2 (20.0%)	0.210
	Male	65 (92.9%)	7 (80.0%)	
Age	<60 years	32 (45.7%)	6 (60.0%)	0.320
	≥60 years	38 (44.4%)	4 (40.0%)	
T classification	T1‐T2	30 (42.9%)		
	T3‐T4	40 (57.1%)		
N classification	N0‐1	55 (78.6%)		
	N2‐3	15 (21.4%)		
Clinical stage	Stage I–II	26 (37.1%)		
	Stage III–IV	44 (62.9%)		
Histologic grade	G1	29 (59.3%)		
	G2	21 (22.2%)		
	G3	20 (18.5%)		
Anatomic site	Oral cavity	15 (29.6%)	3 (30.0%)	0.385
	Oropharynx	4 (3.7%)	2 (20.0%)	
	Hypopharynx	4 (3.7%)	1 (10.0%)	
	Larynx	47 (63.0%)	5 (50.0%)	
KRT13	Negative	29 (41.4)	0	0.042
	Week	12 (17.1)	2 (20)	
	Moderate	16 (22.9)	3 (30)	
	Strong	13 (18.6)	5 (50)	
KRT78	Negative	40 (57.1)	0 (0)	<0.001
	Week	17 (24.3)	3 (30.0)	
	Moderate	8 (12.3)	1 (10.0)	
	Strong	4 (5.7)	6 (60.0)	
SPRR3	Negative	18 (25.7)	0	0.022
	Week	33(47.1)	3 (30.0)	
	Moderate	12 (17.1)	3 (30.0)	
	Strong	7 (10.0)	4 (40.0)	

**FIGURE 12:**
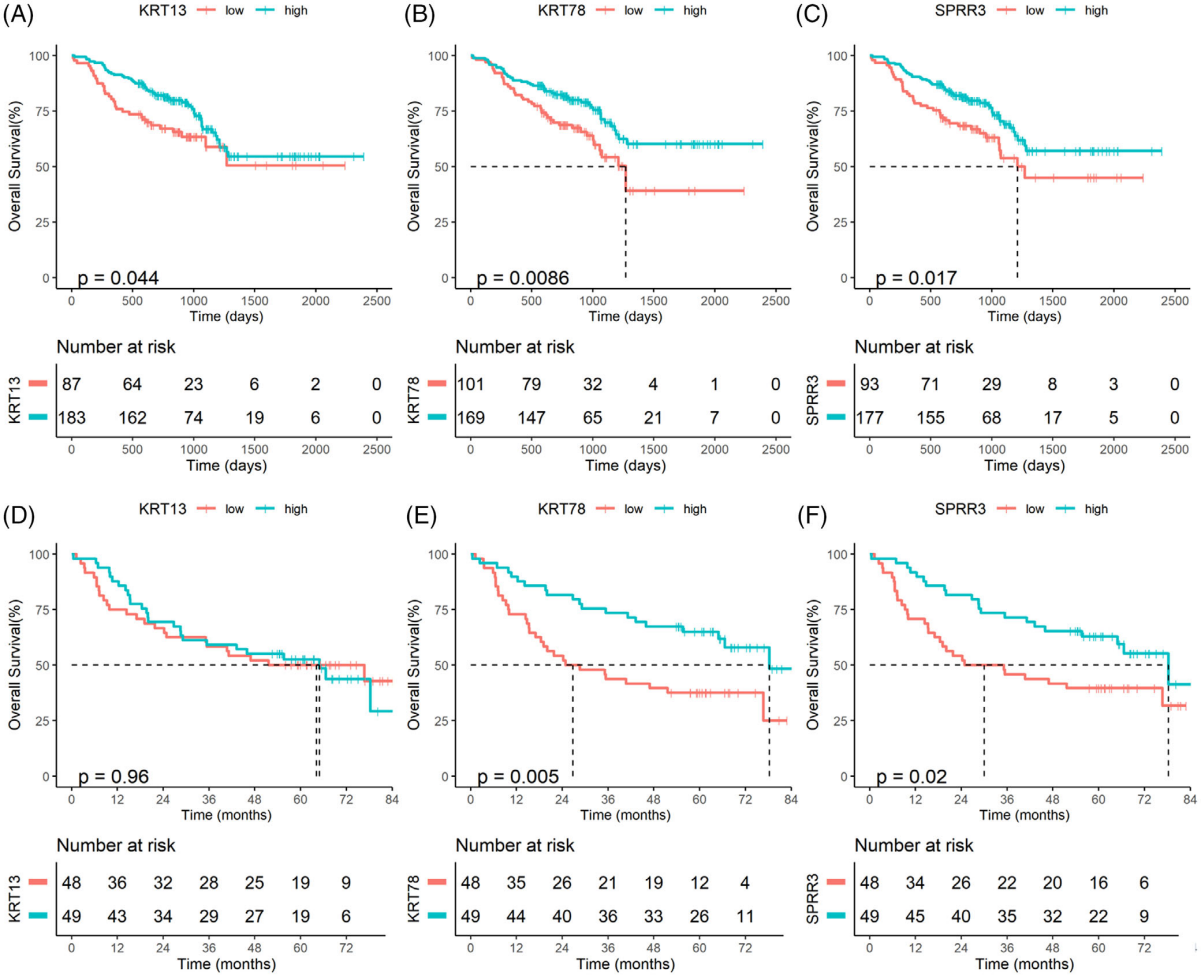
Validation the prognostic value of KRT13, KRT78, and SPRR3 mRNA expression in head and neck squamous cell carcinoma from GSE65858 and GSE41613

After verifying the expression differences of KRT13, KRT78, and SPRR3 in HNSCC and normal tissues, we used GEO datasets (GSE65858 and GSE41613) to analyze their prognostic value (Table 2). In the GSE65858 dataset, survival analysis demonstrated that patients with low KRT13 (*p* = .044), KRT78 (*p* = .0086) and SPRR3 (*p* = .017) expression had worse OS than those with high expression (Figure 12A–C). Furthermore, analysis of the GSE41613 dataset showed that patients with low KRT78 (*p* = .005) and SPRR3 (*p* = .02) expression had worse OS than those with low expression, while no significant difference in OS was found in patients with low and high expression of KRT13 (*p* = .96; Figure 11D–F).

## 4 DISCUSSION

The present study explored the relationship between HNSCC development and immune infiltration via a comprehensive integrated analysis of transcriptomic and clinical data of HNSCC patients in public databases. Ten key genes, namely PPL, SCEL, KRT4, KRT24, KRT78, KRT13, SPRR3, TGM3, CRCT1, and CRNN, were screened by differential expression and PPI network analyses. We found that KRT4, KRT78, and SPRR3 were downregulated in HNSCC and negatively correlated with immune cell infiltration. Furthermore, survival analyses demonstrated that these genes were negatively correlated with OS and PFS in HNSCC. Analysis of the Oncomine and GEO datasets validated that the KRT13, KRT78, and SPRR3 expression levels were downregulated in HNSCC and negatively correlated with the prognosis of patients with HNSCC. Finally, IHC showed that KRT13, KRT78, and SPRR3 protein expression levels were downregulated in HNSCC compared to normal tissues.

Moreover, we demonstrated that 1869 genes and 1578 genes were significantly upregulated and downregulated in HNSCC. Enrichment analysis showed that these DEGs regulate CD4+ T cells, macrophages, and CD8+ T cells. Furthermore, GSEA analysis showed that interferon α and γ reactions were also enriched in HNSCC, suggesting that the immune microenvironment of HNSCC was significantly different from that of normal tissues. It is widely acknowledged that the microenvironment is an essential factor leading to the occurrence and development of tumors,^23^ affecting the efficacy of immunotherapy.^24^, ^25^ Importantly, WGCNA can harness the information of thousands or tens of thousands of the greatest gene expression changes or all genes to identify the gene set of interest and perform significant association analysis with the phenotype.^26^ In the present study, WGCNA showed that out of the 13 modules, the brown module contained the most immune‐related genes (*n* = 7).

PPI network analysis showed that PPL, SCEL, KRT4, KRT24, KRT78, KRT13, SPRR3, TGM3, CRCT1, and CRNN were core components downregulated in HNSCC. KRT4, KRT13, KRT78, and SPRR3 were negatively correlated with CD8+ T cells and macrophage infiltration, suggesting that they mediated the formation of an inhibitory immune micro‐environment.

Interestingly, these four key genes have been documented in keratinization, suggesting that keratinization not only reflects the cell differentiation level but also determines the immune state.

Besides, we also revealed that the expression levels of KRT4, KRT78, KRT13, and SPRR3 in TCGA‐HNSCC negatively correlated with immune cell infiltration and influenced patient prognosis. Analysis of the two Oncomine datasets also confirmed that KRT13, KRT78, and SPRP3 expression levels were significantly lower in HNSCC than in normal tissues. In addition to the significant reduction in mRNA expression levels of KRT13, KRT78, and SPRR3 in HNSCC, we also documented a significant decline in the protein expression levels of KRT13, KRT78, and SPRR3 in HNSCC using IHC. This means that these 3 genes are related to the immune infiltration and prognosis of NPC, and they are potential immune‐related genes. Many previous studies had found that multiple immune‐related genes were associated with the prognosis of NPC. These genes were known immune‐related genes, such as CCR5, CD3E, CD4, SFRP4, SFRP4, CPXM1, and COL5A1 CCR6, CCL22, ROBO1, DKK1 and PDGFA, and so forth.^11^, ^27^, ^28^ In our study, WGCNA was used to lock immune‐related modules and then protein–protein interaction network was used to screen out potential immune‐related genes. The subsequent analysis also found that KRT13, KRT78, and SPRP3 were negatively correlated with immune cell infiltration. KRT13, KRT78, and SPRP3 are newly discovered immune‐related genes in HNSCC in our study.Herein, we found that patients with low expression of KRT13, KRT78, and SPRR3 in the TCGA‐HNSCC dataset had worse OS and PFI than those with high expression. This finding was also externally validated in two public GEO datasets (GSE65858 and GSE41613), where the low expression of KRT78 and SPRR3 in HNSCC patients was associated with poor OS. Our results indicated that the main biological function of Keratin 78 and SPRP3 is to participate in developmental biology and cell keratinization. Hence, these two key genes can inhibit the occurrence and progression of HNSCC acting as tumor suppressor genes. Although preliminary studies have shown that KRT78^29^ and SPRR3^30^ are involved in cytokeratinization, their role in tumors remains unknown. Current evidence suggests that SPRR3 expression is low in HNSCC^26^ and esophageal cancer,^31^ while other studies have substantiated that SPRR3 is a prognostic factor of esophageal cancer and a sensitive marker of chemotherapy and radiotherapy.^32^, ^33^, ^34^ At present, few reports have shown that KRT78 is differentially expressed in melanoma,^35^ colon adenocarcinoma,^36^ cervical cancer,^37^ urinary bladder cancer,^38^ and HNSCC,^39^ and may be related to tumor prognosis.^37^, ^38^ However, the biological function of KRT78 in tumors remains primarily understudied, warranting further in‐depth studies.

Although this study enhanced our understanding of the involvement of KRT78 and SPRR3 in HNSCC, there were some limitations and shortcomings. First, the detailed mechanisms underlying the KRT78‐ and SPRR3‐mediated decrease in immune cell infiltration and biological functions in HNSCC were not explored. Indeed, in vivo and in vitro experiments are needed to explore and verify their biological functions. Although our study confirmed that KRT13, KRT78, and SPRR3 protein expression levels in HNSCC were significantly downregulated, the prognostic value of protein expression could not be analyzed due to the lack of survival data. It is necessary to conduct a perspective study to demonstrate the prognostic value of KRT13, KRT78, and SPRR3 protein expression.

To conclude, we identified four hitherto unrecognized key genes, KRT4, KRT78, KRT13, and SPRR3, related to the occurrence and development of HNSCC and positively correlated with immune cell infiltration. KRT78 and SPRR3, novel immune‐related genes, might serve as diagnostic and prognostic biomarkers of HNSCC. Nonetheless, the detailed biological functions and clinical value of KRT78 and SPRR3 in HNSCC need further exploration.

## AUTHOR CONTRIBUTIONS

**Qiaojuan Guo:** Conceptualization (equal); investigation (equal); writing – original draft (equal); writing – review and editing (equal). **Tianzhu Lu:** Formal analysis (equal); writing – original draft (equal). **Hanchuan Xu:** Formal analysis (supporting); investigation (supporting); writing – original draft (supporting). **Qingfeng Luo:** Data curation (supporting); formal analysis (supporting). **Zhilang Liu:** Data curation (supporting); formal analysis (supporting). **Sicong Jiang:** Date curation(supporting); formal analysis (supporting). **Shao jun Lin:** Writing – original draft (supporting). **Jianji Pan:** Writing – original draft (supporting); writing – review and editing (supporting). **Mengyao Lin:** Formal analysis (supporting); writing – original draft (supporting); writing – review and editing (supporting). **Fang Guo:** Conceptualization (lead); writing – original draft (lead); writing – review and editing (lead).

## CONFLICT OF INTEREST STATEMENT

The authors declare no conflict of interest.

## ETHICS STATEMENT

This study was approved by the Hospital Review Board of Fujian Cancer Hospital, Fujian, China. The Review Board Committee granted a waiver of informed consent for this study.

## Supporting information


**Table S1.** The top 10 UP‐regulated and Down‐regulated differentially expressed genes in Head and neck cancer (selected according to the ascending order of P value)
**Table S2.** GO and immune characteristic function enrichment analysis information based on differentially expressed genes.
**Table S3.** GSEA analysis in HNSCC.
**Table S3.** List of immune‐related DEGs in Brown module

## Data Availability

The datasets used and/or analysed during the current study are available from the corresponding author on reasonable request.
