# Supplementary material for: Identification of immune‐related genes contributing to head and neck squamous cell carcinoma development using weighted gene co‐expression network analysis
Source: Cancer Rep (Hoboken). 2023 Apr 24;6(5):e1808. doi: 10.1002/cnr2.1808 (PMC10172170; doi:10.1002/cnr2.1808)
Supplement: Supplementary file 1 — Table S1. The top 10 UP‐regulated and Down‐regulated differentially expressed genes in Head and neck cancer (selected according to the ascending order of P value) Table S2. GO and immune characteristic function enrichment analysis information based on differentially expressed genes. Table S3. GSEA analysis in HNSCC. Table S3. List of immune‐related DEGs in Brown module [file CNR2-6-e1808-s001.docx]

**Supplementary Table 1.** **The top 10 UP-regulated and Down-regulated** **differentially expressed genes in Head and neck cancer (selected according to the ascending order of P value)**

| **Up-regulated genes in HNSC (Top10)** | | | |
| --- | --- | --- | --- |
| **Ensembl ID** | **Gene symbol** | **log2(fold change)** | **P value** |
| ENSG00000107159 | CA9 | 5.849712962 | 4.21E-82 |
| ENSG00000099953 | MMP11 | 5.075474217 | 9.94E-75 |
| ENSG00000095752 | IL11 | 4.317418163 | 3.66E-70 |
| ENSG00000123500 | COL10A1 | 5.349082475 | 4.07E-65 |
| ENSG00000250133 | HOXC-AS2 | 3.927130231 | 1.49E-63 |
| ENSG00000248554 | C5orf34-AS1 | 3.349280768 | 7.35E-62 |
| ENSG00000197757 | HOXC6 | 3.706884422 | 3.07E-60 |
| ENSG00000237424 | FOXD2-AS1 | 2.392821346 | 7.39E-60 |
| ENSG00000169258 | GPRIN1 | 2.098282013 | 7.88E-58 |
| ENSG00000261327 | AC134312.5 | 4.163823415 | 4.32E-57 |
| **Down-regulated genes in HNSC (Top10)** | | | |
| ENSG00000231887 | PRH1 | -7.414327992 | 1.59E-113 |
| ENSG00000106351 | AGFG2 | -2.371015265 | 6.17E-82 |
| ENSG00000070081 | NUCB2 | -2.145545345 | 1.38E-81 |
| ENSG00000102547 | CAB39L | -2.210238442 | 7.50E-78 |
| ENSG00000151882 | CCL28 | -4.178352088 | 1.87E-75 |
| ENSG00000163072 | NOSTRIN | -2.489489093 | 9.94E-75 |
| ENSG00000152642 | GPD1L | -2.751197749 | 3.58E-72 |
| ENSG00000227028 | SLC8A1-AS1 | -4.890511751 | 1.80E-71 |
| ENSG00000134531 | EMP1 | -3.187895183 | 3.70E-71 |
| ENSG00000101441 | CST4 | -8.130098448 | 9.53E-70 |

**Supplementary Table 2. GO and immune characteristic function enrichment analysis information based on differentially expressed genes.**

| **Biological Process** | | | | | |
| --- | --- | --- | --- | --- | --- |
| **GO** | **Description** | **Count** | **%** | **Log10(P)** | **Log10(q)** |
| GO:0006936 | muscle contraction | 93 | 5.09 | -26.24 | -22.04 |
| GO:0030239 | myofibril assembly | 34 | 1.86 | -20.03 | -16.53 |
| GO:0061061 | muscle structure development | 123 | 6.74 | -19.77 | -16.34 |
| GO:0001501 | skeletal system development | 96 | 5.26 | -15.44 | -12.38 |
| GO:0043269 | regulation of ion transport | 115 | 6.3 | -15.08 | -12.06 |
| GO:0043062 | extracellular structure organization | 82 | 4.49 | -14.57 | -11.6 |
| GO:0060047 | heart contraction | 63 | 3.45 | -14.43 | -11.5 |
| GO:0030855 | epithelial cell differentiation | 119 | 6.52 | -12.98 | -10.26 |
| GO:0098662 | Inorganic cation transmembrane transport | 110 | 6.02 | -10.54 | -7.97 |
| GO:0048729 | tissue morphogenesis | 99 | 5.42 | -9.91 | -7.41 |
| GO:0060415 | muscle tissue morphogenesis | 25 | 1.37 | -9.4 | -6.96 |
| GO:0010817 | regulation of hormone levels | 81 | 4.44 | -8.94 | -6.54 |
| GO:0051216 | cartilage development | 43 | 2.35 | -8.78 | -6.39 |
| GO:0043501 | skeletal muscle adaptation | 14 | 0.77 | -8.61 | -6.24 |
| GO:0019730 | antimicrobial humoral response | 30 | 1.64 | -8.24 | -5.92 |
| GO:0042391 | regulation of membrane potential | 68 | 3.72 | -7.91 | -5.63 |
| GO:0006820 | anion transport | 88 | 4.82 | -7.76 | -5.51 |
| GO:0010466 | negative regulation of peptidase activity | 47 | 2.57 | -7.52 | -5.29 |
| GO:0042445 | hormone metabolic process | 43 | 2.35 | -7.06 | -4.86 |
| GO:0014866 | skeletal myofibril assembly | 8 | 0.44 | -6.88 | -4.71 |
| **Molecular Function** | | | | | |
| GO:0005198 | structural molecule activity | 137 | 7.5 | -24.25 | -20.58 |
| GO:0008307 | structural constituent of muscle | 27 | 1.48 | -19.55 | -16.36 |
| GO:0048018 | receptor ligand activity | 92 | 5.04 | -16.36 | -13.3 |
| GO:0003779 | actin binding | 81 | 4.44 | -13.98 | -11.09 |
| GO:0022803 | passive transmembrane transporter activity | 81 | 4.44 | -12.54 | -9.72 |
| GO:0030020 | extracellular matrix structural constituent conferring tensile strength | 20 | 1.1 | -11.79 | -9.12 |
| GO:0005509 | calcium ion binding | 105 | 5.75 | -11.07 | -8.45 |
| GO:0030414 | peptidase inhibitor activity | 42 | 2.3 | -10.11 | -7.56 |
| GO:0005539 | glycosaminoglycan binding | 47 | 2.57 | -9.44 | -6.98 |
| GO:0015108 | chloride transmembrane transporter activity | 25 | 1.37 | -7.12 | -4.9 |
| GO:0001228 | DNA-binding transcription activator activity, RNA polymerase II-specific | 64 | 3.5 | -6.76 | -4.61 |
| GO:0042805 | actinin binding | 14 | 0.77 | -6.54 | -4.4 |
| GO:0008509 | anion transmembrane transporter activity | 50 | 2.74 | -6.07 | -3.99 |
| GO:0019825 | oxygen binding | 13 | 0.71 | -5.99 | -3.93 |
| GO:0051373 | FATZ binding | 5 | 0.27 | -5.63 | -3.62 |
| GO:0005179 | hormone activity | 25 | 1.37 | -5.47 | -3.47 |
| GO:0020037 | heme binding | 25 | 1.37 | -4.67 | -2.69 |
| GO:1901618 | organic hydroxy compound transmembrane transporter activity | 13 | 0.71 | -4.55 | -2.6 |
| GO:0044325 | ion channel binding | 24 | 1.31 | -4.54 | -2.6 |
| GO:0008188 | neuropeptide receptor activity | 13 | 0.71 | -4.44 | -2.51 |
| **Cellular Component** | | | | | |
| GO:0043292 | contractile fiber | 95 | 5.2 | -44.78 | -41.49 |
| GO:0031012 | extracellular matrix | 116 | 6.35 | -25.22 | -22.71 |
| GO:0031672 | A band | 24 | 1.31 | -17.93 | -15.6 |
| GO:0042383 | sarcolemma | 44 | 2.41 | -17.21 | -14.92 |
| GO:0044420 | extracellular matrix component | 24 | 1.31 | -12.88 | -10.71 |
| GO:0005865 | striated muscle thin filament | 16 | 0.88 | -12 | -9.89 |
| GO:1902495 | transmembrane transporter complex | 62 | 3.4 | -11.38 | -9.32 |
| GO:0016528 | sarcoplasm | 27 | 1.48 | -10.97 | -8.96 |
| GO:0005788 | endoplasmic reticulum lumen | 59 | 3.23 | -10.71 | -8.74 |
| GO:0032982 | myosin filament | 14 | 0.77 | -10.52 | -8.57 |
| GO:0016324 | apical plasma membrane | 56 | 3.07 | -7.93 | -6.1 |
| GO:0005882 | intermediate filament | 41 | 2.25 | -7.65 | -5.89 |
| GO:0031225 | anchored component of membrane | 35 | 1.92 | -7.65 | -5.89 |
| GO:0034707 | chloride channel complex | 15 | 0.82 | -5.64 | -3.95 |
| GO:0045211 | postsynaptic membrane | 43 | 2.35 | -5.14 | -3.47 |
| GO:0014704 | intercalated disc | 14 | 0.77 | -4.81 | -3.16 |
| GO:0030424 | axon | 75 | 4.11 | -4.18 | -2.55 |
| GO:0098642 | network-forming collagen trimer | 5 | 0.27 | -3.96 | -2.35 |
| GO:1904724 | tertiary granule lumen | 13 | 0.71 | -3.78 | -2.18 |
| GO:0005796 | Golgi lumen | 19 | 1.04 | -3.73 | -2.14 |
| **Immunologic Signature** | | | | | |
| M8465 | WT vs mir17 overexpression act CD4 T cell DN | 62 | 3.4 | -22.08 | -18.39 |
| M10033 | Spleen macrophage vs colon macrophage DN | 55 | 3.01 | -17.12 | -13.73 |
| M5307 | Yong vs. old donor memory CD4 T cell DN | 45 | 2.46 | -10.88 | -7.67 |
| M7508 | Tfh vs non Tfh CD4 T cell DN | 44 | 2.41 | -10.16 | -7.07 |
| M7345 | Adenosine A3R inh vs. inh pretreat and with T cell membranes mast cell DN | 43 | 2.35 | -9.75 | -6.77 |
| M4845 | CD8 T cell vs erythroblast DN | 43 | 2.35 | -9.68 | -6.77 |
| M8800 | C57BL6 vs non Foxp3 fusion GFP treg UP | 29 | 1.59 | -9.57 | -6.73 |
| M3306 | Day3 vs Day21 yf17d vaccine PBMC DN | 41 | 2.25 | -9.47 | -6.68 |
| M9430 | In vivo nTreg vs in vitro iTreg UP | 41 | 2.25 | -8.68 | -5.94 |
| M4514 | IL2 vs IL15 stim NK cell UP | 40 | 2.19 | -8.29 | -5.6 |
| M4985 | pre vs day7 post tiv flu vaccine B cell UP | 40 | 2.19 | -8.23 | -5.58 |
| M4297 | naïve vs IL2 ralow day3 EFF CD8 T cell UP | 40 | 2.19 | -8.03 | -5.46 |
| M4978 | Pre vs day7 flu vaccine pdc UP | 40 | 2.19 | -8.03 | -5.46 |
| M7766 | WT vs myd88 ko macrophage 48h mbovis BCG stim UP | 39 | 2.14 | -7.59 | -5.05 |
| M433 | Health vs peridontis neutrophils | 30 | 1.64 | -7.49 | -4.97 |
| M470 | unstim vs IFNB stim raw264 cells DN | 27 | 1.48 | -7.37 | -4.89 |
| M2913 | wt vs IFNG ko skin UP | 28 | 1.53 | -7.29 | -4.84 |
| M7843 | IL35 Treated vs resting Treg DN | 38 | 2.08 | -7.05 | -4.61 |
| M9812 | UP Unstim vs CD40L IL12 IL15 3day stimulated IRF4 KO B cell UP | 36 | 1.97 | -6.92 | -4.51 |
| M8361 | Mautral vs induced Treg UP | 37 | 2.03 | -6.8 | -4.42 |

**Supplementary Table 3. GSEA analysis in HNSCC.**

| **Enrichment in Normal group** | | | | | | | |
| --- | --- | --- | --- | --- | --- | --- | --- |
| OXIDATIVE_PHOSPHORYLATION | 200 | 0.685 | 3.148 | 0 | 0 | 0 | 8788 |
| ADIPOGENESIS | 199 | 0.642 | 2.963 | 0 | 0 | 0 | 8160 |
| MYOGENESIS | 199 | 0.591 | 2.732 | 0 | 0 | 0 | 6429 |
| BILE_ACID_METABOLISM | 112 | 0.631 | 2.708 | 0 | 0 | 0 | 7333 |
| FATTY_ACID_METABOLISM | 158 | 0.592 | 2.617 | 0 | 0 | 0 | 8703 |
| ESTROGEN_RESPONSE_EARLY | 199 | 0.519 | 2.407 | 0 | 0 | 0 | 6621 |
| PEROXISOME | 104 | 0.547 | 2.325 | 0 | 0 | 0 | 7216 |
| XENOBIOTIC_METABOLISM | 200 | 0.451 | 2.08 | 0 | 0 | 0 | 7100 |
| HEME_METABOLISM | 199 | 0.441 | 2.022 | 0 | 0 | 0 | 8355 |
| KRAS_SIGNALING_DN | 199 | 0.41 | 1.884 | 0 | 0.000122 | 0.001 | 4460 |
| ANDROGEN_RESPONSE | 100 | 0.462 | 1.882 | 0 | 0.000111 | 0.001 | 7510 |
| UV_RESPONSE_DN | 144 | 0.422 | 1.86 | 0 | 0.000102 | 0.001 | 6867 |
| ESTROGEN_RESPONSE_LATE | 199 | 0.385 | 1.77 | 0 | 0.00111 | 0.01 | 4743 |
| REACTIVE_OXYGEN_SPECIES_PATHWAY | 49 | 0.477 | 1.751 | 0 | 0.00112 | 0.011 | 6475 |
| PROTEIN_SECRETION | 96 | 0.374 | 1.54 | 0.00303 | 0.00772 | 0.074 | 5485 |
| P53_PATHWAY | 200 | 0.332 | 1.537 | 0 | 0.00724 | 0.074 | 8360 |
| KRAS_SIGNALING_UP | 200 | 0.297 | 1.362 | 0.0246 | 0.0342 | 0.346 | 6850 |
| TNFA_SIGNALING_VIA_NFKB | 200 | 0.289 | 1.347 | 0.0106 | 0.0372 | 0.385 | 8479 |
| CHOLESTEROL_HOMEOSTASIS | 74 | 0.321 | 1.288 | 0.076 | 0.06 | 0.561 | 11561 |
| COAGULATION | 138 | 0.29 | 1.265 | 0.0392 | 0.0697 | 0.625 | 6326 |
| **Enrichment in HNSC group** | | | | | | | |
| E2F_TARGETS | 200 | -0.778 | -3.290 | 0 | 0 | 0 | 3703 |
| G2M_CHECKPOINT | 200 | -0.719 | -3.051 | 0 | 0 | 0 | 3424 |
| INTERFERON_ALPHA_RESPONSE | 97 | -0.726 | -2.762 | 0 | 0 | 0 | 6069 |
| EPITHELIAL_MESENCHYMAL_TRANSITION | 200 | -0.646 | -2.734 | 0 | 0 | 0 | 4958 |
| INTERFERON_GAMMA_RESPONSE | 200 | -0.606 | -2.609 | 0 | 0 | 0 | 6534 |
| MYC_TARGETS_V1 | 200 | -0.548 | -2.325 | 0 | 0 | 0 | 9077 |
| DNA_REPAIR | 150 | -0.535 | -2.172 | 0 | 0 | 0 | 6754 |
| MITOTIC_SPINDLE | 199 | -0.493 | -2.086 | 0 | 0 | 0 | 4480 |
| ANGIOGENESIS | 36 | -0.649 | -2.082 | 0 | 0 | 0 | 6148 |
| MYC_TARGETS_V2 | 58 | -0.574 | -2.035 | 0 | 1.00E-04 | 0.001 | 10942 |
| ALLOGRAFT_REJECTION | 200 | -0.471 | -1.988 | 0 | 9.09E-05 | 0.001 | 10563 |
| MTORC1_SIGNALING | 199 | -0.460 | -1.918 | 0 | 8.33E-05 | 0.001 | 9006 |
| APICAL_JUNCTION | 199 | -0.399 | -1.679 | 0 | 0.00174 | 0.028 | 7845 |
| GLYCOLYSIS | 199 | -0.397 | -1.674 | 0 | 0.00167 | 0.029 | 5711 |
| UV_RESPONSE_UP | 158 | -0.407 | -1.668 | 0 | 0.00172 | 0.032 | 6424 |
| UNFOLDED_PROTEIN_RESPONSE | 112 | -0.423 | -1.652 | 0.00447 | 0.00208 | 0.041 | 9445 |
| INFLAMMATORY_RESPONSE | 199 | -0.389 | -1.641 | 0 | 0.00223 | 0.047 | 9166 |
| IL6_JAK_STAT3_SIGNALING | 87 | -0.430 | -1.632 | 0.00298 | 0.00224 | 0.05 | 5407 |
| TGF_BETA_SIGNALING | 54 | -0.439 | -1.496 | 0.0182 | 0.01132 | 0.235 | 5413 |
| SPERMATOGENESIS | 134 | -0.363 | -1.477 | 0.00285 | 0.0130 | 0.277 | 3424 |

**Supplementary Table 3**. List of immune-related DEGs in Brown module

| **Gene symbol** | **Chromosome** | **Start** | **End** | **Strand** | **Description** |
| --- | --- | --- | --- | --- | --- |
| CEACAM1 | chr19 | 42507304 | 42561234 | -1 | CEA cell adhesion molecule 1 |
| IL1RN | chr2 | 113107214 | 113134016 | 1 | interleukin 1 receptor antagonist |
| IL36A | chr2 | 113005459 | 113008044 | 1 | interleukin 36 alpha |
| A2ML1 | chr12 | 8822621 | 8887001 | 1 | alpha-2-macroglobulin like 1 |
| FUT3 | chr19 | 5842888 | 5851474 | -1 | fucosyltransferase 3 (Lewis blood group) |
| FCER1A | chr1 | 159289714 | 159308224 | 1 | Fc fragment of IgE receptor Ia |
| CXCR2 | chr2 | 218125289 | 218137251 | 1 | C-X-C motif chemokine receptor 2 |
